# Serum *N*‐glycome alterations in breast cancer during multimodal treatment and follow‐up

**DOI:** 10.1002/1878-0261.12105

**Published:** 2017-07-24

**Authors:** Radka Saldova, Vilde D. Haakensen, Einar Rødland, Ian Walsh, Henning Stöckmann, Olav Engebraaten, Anne‐Lise Børresen‐Dale, Pauline M. Rudd

**Affiliations:** ^1^ NIBRT GlycoScience Group National Institute for Bioprocessing Research and Training Dublin Ireland; ^2^ Department of Cancer Genetics Institute for Cancer Research Oslo University Hospital The Norwegian Radium Hospital Norway; ^3^ Department of Tumor Biology Institute for Cancer Research Oslo University Hospital The Norwegian Radium Hospital Norway; ^4^ Bioprocessing Technology Institute Agency for Science, Technology and Research (A*STAR) Singapore Singapore; ^5^ Department of Oncology Oslo University Hospital Norway; ^6^ Institute for Clinical Medicine University of Oslo Norway; ^7^ Present address: AbbVie Inc. 1 North Waukegan Road North Chicago IL 60064 USA

**Keywords:** breast cancer, CRP, follow‐up, inflammation, serum *N*‐glycans, treatment

## Abstract

Using our recently developed high‐throughput automated platform, *N*‐glycans from all serum glycoproteins from patients with breast cancer were analysed at diagnosis, after neoadjuvant chemotherapy, surgery, radiotherapy and up to 3 years after surgery. Surprisingly, alterations in the serum *N*‐glycome after chemotherapy were pro‐inflammatory with an increase in glycan structures associated with cancer. Surgery, on the other hand, induced anti‐inflammatory changes in the serum *N*‐glycome, towards a noncancerous phenotype. At the time of first follow‐up, glycosylation in patients with affected lymph nodes changed towards a malignant phenotype. C‐reactive protein showed a different pattern, increasing after first line of neoadjuvant chemotherapy, then decreasing throughout treatment until 1 year after surgery. This may reflect a switch from acute to chronic inflammation, where chronic inflammation is reflected in the serum after the acute phase response subsides. In conclusion, we here present the first time‐course serum *N*‐glycome profiling of patients with breast cancer during and after treatment. We identify significant glycosylation changes with chemotherapy, surgery and follow‐up, reflecting the host response to therapy and tumour removal.

**Abbreviations**
18F‐FDG PET/CT: 2‐deoxy‐2‐[fluorine‐18]fluoro‐d‐glucose integrated with computed tomography
2‐AB: 2‐aminobenzamide
CRP: C‐reactive protein
ER: estrogen receptor
FDR: false discovery rate
FEC: 5‐fluorouracil, epirubicin and cyclophosphamide
HER‐2: human epidermal growth factor receptor‐2
HILIC: hydrophilic interaction liquid chromatography
HNFα: hepatocyte nuclear factor 1α
PNGase F: peptide *N*‐glycanase F
PR: progesterone receptor
sLex: sialyl Lewis x epitope
UPLC: ultra‐performance liquid chromatography

## 1 Introduction

Breast cancer is the most common cancer among females in the Western world and leading cause of cancer‐related death among women worldwide (Torre *et al*., 2016).

Breast cancer treatment options include surgery, chemotherapy, endocrine therapies and radiotherapy (Bethesda, 2016; Tang *et al*., 2016). Neoadjuvant chemotherapy is given to reduce the tumour size before surgery. In this study, the patients were given neoadjuvant FEC (5‐fluorouracil, epirubicin and cyclophosphamide) followed by paclitaxel. Bevacizumab is a humanized monoclonal antibody directed against vascular endothelial growth factor A, but its role in the treatment for metastatic breast cancer remains controversial (Bethesda, 2016). Patients were randomized to bevacizumab or not. Radiotherapy follows after breast‐conserving surgery to eradicate residual disease reducing risk of recurrence (Bethesda, 2016).

Unfortunately, many breast carcinomas are resistant to treatment (Tang *et al*., 2016). Monitoring response in patients with breast cancer receiving neoadjuvant chemotherapy would be beneficial to identify patients who need less or more aggressive treatment and who respond to chemotherapy. Techniques for monitoring effect of treatment include clinical measurement by calliper (Snelling *et al*., 2004), magnetic resonance imaging (MRI) (Prevos *et al*., 2012), positron emission tomography with 2‐deoxy‐2‐[fluorine‐18]fluoro‐d‐glucose integrated with computed tomography (Avril *et al*., 2016).

Glycosylation is the most common post‐translational modification of proteins, and changes in glycosylation have been described in many diseases such as cancer, chronic inflammatory diseases and genetic glycosylation disorders (Marino *et al*., 2012; Pinho and Reis, 2015). The most significant cancer‐associated changes in glycosylation are sialylation, fucosylation, *O*‑glycan truncation and *N*‑ and *O*‑linked glycan branching (Pinho and Reis, 2015). This altered expression of glycans can be due to under‐ or overexpression and localization of glycosyltransferases, changes in the tertiary conformation of the peptide backbone and that of the nascent glycan chain, variability of various acceptor substrates or the availability and abundance of the sugar nucleotide donors and cofactors (Pinho and Reis, 2015). Glycans have been found to participate in many basic biological processes in cancer, such as inflammation, immune surveillance, cell–cell adhesion, cell–matrix interaction, inter‐ and intracellular signalling and cellular metabolism (Pinho and Reis, 2015).

The role of glycosylation in breast cancer was described in relation to the formation of metastasis, cancer progression, diagnostics and therapy (Kolbl *et al*., 2015). In our previous studies, we found significant changes in glycosylation including sialylation, fucosylation, branching and content of high mannosylated glycans in breast cancer, indicating the presence of metastasis, spread to the lymph nodes and correlation with tumour circulating cells, tumour characteristics such as TP53 mutation or hormone status and systemic features such as oestradiol levels, BMI and mammographic density (Marino *et al*., 2012; Saldova *et al*., 2014). Increases in sialylation, branching, outer arm fucose [which is part of sialyl Lewis x epitope (sLex)] and decrease in high mannosylated and biantennary core‐fucosylated glycans were found in patients with breast cancer compared with healthy controls (Marino *et al*., 2012; Saldova *et al*., 2014). Levels of the sLex increased with metastasis and with higher number of circulating tumour cells in advanced‐stage patients and were found to be increased together with core‐fucosylated agalactosylated biantennary glycans in lymph node‐positive compared with lymph node‐negative early‐stage breast cancer patients (Marino *et al*., 2012). Bisected biantennary nonfucosylated glycans decreased in patients with progesterone receptor‐positive tumours, and core‐fucosylated biantennary bisected monogalactosylated glycans decreased in patients with the TP53 mutation (Saldova *et al*., 2014). Serum oestradiol was associated with increase in digalactosylated glycans, and higher mammographic density was associated with an increase in biantennary digalactosylated glycans and with a decrease in trisialylated and in outer arm fucosylated glycans (Saldova *et al*., 2014).

Few publications looked at serum glycosylation in relation to cancer treatment monitoring. Glycosylation on serum IgG and IgM was found not to be changed after tumour ablation (liver, renal and lung tumours) (Breen *et al*., 2016). Miyahara *et al*. (2015) described high mannosylated glycan Man9 as potential serum *N*‐glycan biomarker of gemcitabine treatment efficacy in patients with pancreatic cancer; high levels of Man9 were correlated with short time to tumour progression and overall survival. We have found increases in sialylation and branching correlated with inflammation, pro‐inflammatory drug treatment and ovarian tumour progression in mouse model (Saldova *et al*., 2013).

Methods to analyse glycome include NMR, capillary electrophoresis, lectin capturing, liquid chromatography fluorescence and mass spectrometric methods (Clerc *et al*., 2016; Etxebarria and Reichardt, 2016; Marino *et al*., 2010). A high‐throughput automated platform for *N*‐glycan analysis of human serum using ultra‐performance liquid chromatography (UPLC)–hydrophilic interaction liquid chromatography (HILIC) with fluorescence detection was described previously (Saldova *et al*., 2014; Stöckmann *et al*., 2015). HILIC is based on hydrophilic potential of the glycan, affected by its size, charge, composition, structure, linkage and oligosaccharide branching, and when labelled with 2‐aminobenzamide (2‐AB), it enables accurate quantitative measurements and comparison between samples (Marino *et al*., 2010).

Using our high‐throughput automated platform to analyse *N‐*glycans from breast cancer patient samples before, during and after cancer treatment, we describe associated changes in serum *N*‐glycome and discuss the possible underlying reason.

## 2 Materials and methods

### 2.1 Clinical information and collection of serum samples

Women receiving neoadjuvant treatment for breast cancer at Oslo University Hospital, Oslo, Norway, and at St Olavs Hospital, Trondheim, Norway, were recruited to this study. Written informed consents were obtained from all patients prior to inclusion. The study was approved by the institutional protocol review board, the regional ethics committee, the Norwegian Medicines Agency and carried out in accordance with the Declaration of Helsinki, International Conference on Harmony/Good Clinical Practice. The study is registered in the http://www.ClinicalTrials.gov/ database with the identifier NCT00773695. Chemotherapy was given as standard including four cycles of FEC [fluorouracil (5‐FU), epirubicin and cyclophosphamide] followed by 12‐week treatment with taxane (docetaxel given once every third week or weekly administration of paclitaxel). (Twelve women with estrogen receptor (ER)‐positive breast cancer received neoadjuvant endocrine therapy in a separate cohort of the trial.) The patients given chemotherapy were randomized to bevacizumab (*n* = 67) or chemotherapy only (*n* = 71). One of the patients randomized to receive chemotherapy was found ineligible for study entry and did not receive treatment. In the patients receiving bevacizumab and chemotherapy, two discontinued treatment due to metastatic disease, two patients experienced cardiac and neurological side effects and one patient died from unknown cause. All patients receiving any treatment were included in the safety analyses (see Silwal‐Pandit *et al*., 2017), and 66 patients in each arm were evaluated for efficacy. The amount of remaining tumour tissue and final response to therapy were determined by the pathologist after surgery: the primary endpoint was pathological complete response, defined as complete eradication of all invasive cancer cells in both breast and axillary lymph nodes. For other patients, the degree of response was determined by the calculation of tumour shrinkage from initial measurements made before treatment start, using MRI if available for the patients. Otherwise, ultrasound or mammography measurements were taken. Patients were defined as having a (partial) response if the tumour diameter was 30% or less compared to the initial measurements. Summary of patients and their clinical characteristics is shown in Table 1.

**Table 1:** Total numbers of the women included in the study

Clinical features	Categories	No	%
Age	< 50	68	47
	50–< 60	49	34
	60–< 70	24	17
	70+	3	2
pN	0	53	37
	1–3	59	41
	NA	32	22
pT	< 2 cm	48	33
	2–< 5 cm	51	35
	> 5 cm	20	14
	NA	25	17
ER	Neg	23	16
	Pos	121	84
Bevacizumab use	No	73	51
	Yes	71	49
Endocrine	No	132	92
	Yes	12	8
Response	Complete	21	15
	Partial	76	53
	Nonresponders	38	26
	NA	9	6

**Figure 1:**
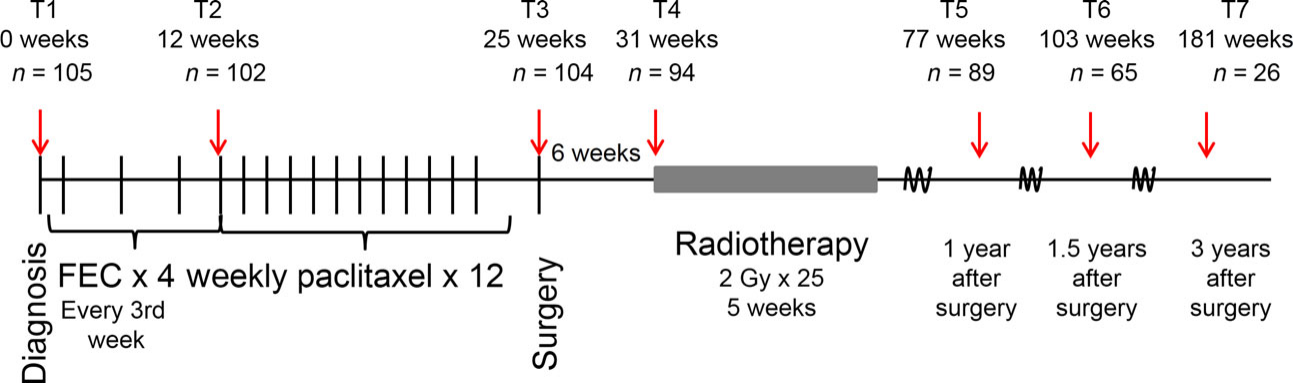
A flow diagram describing when the blood samples were taken.

Serum samples were collected from the patients at the time of diagnosis (T1), after four cycles of FEC (T2), after completion of taxane treatment (T3), before the start of radiotherapy (6 weeks after surgery) (T4), 1 year postsurgery (T5), 1.5 years postsurgery (T6) and 3 years postsurgery (T7) (Fig. 1). Serum was collected in tubes without gel, left for 30–60 min before centrifugation at 2900 rpm for 10 min and stored at −80 °C.

### 2.2 *N*‐glycan analyses

*N*‐glycans were released from 5 μL of serum samples using the high‐throughput automated method previously described by Stöckmann *et al*. (2015). Briefly, the samples were denatured with dithiothreitol, alkylated with iodoacetamide, and *N*‐glycans were released from the protein backbone enzymatically via PNGase F (Prozyme peptide *N*‐glycanase F, code GKE‐5006D, 10 μL per well, 0.5 mU in 1 m ammonium bicarbonate, pH 8.0). Next, glycans were immobilized on solid supports, and excess reagents were removed by vacuum or centrifuge filtration. Glycans were released from the solid supports and labelled with the fluorophore 2‐AB. Next, glycans were cleaned up using 96‐well chemically inert filter plate (Millipore Solvinert, hydrophobic polytetrafluoroethylene membrane, 0.45 μm pore size) using HyperSepDiol SPE cartridges (ThermoScientific, Waltham, MA, USA) (Stockmann *et al*., 2013).

### 2.3 Ultra‐performance liquid chromatography (UPLC)

2.1 × 150 mm column (Waters, Milford, MA, USA) on an Acquity UPLC (Waters) equipped with a Waters temperature control module and a Waters Acquity fluorescence detector was used. Solvent A was 50 mm formic acid adjusted to pH 4.4 with ammonia solution. Solvent B was acetonitrile. The column temperature was set to 40 °C. The 30‐min method was used with a linear gradient of 30–47% with buffer A at 0.56 mL·min^−1^ flow rate for 23 min followed by 47–70% A and finally reverting back to 30% A to complete the run method. Samples were injected in 70% acetonitrile. Fluorescence was measured at 420 nm with excitation at 330 nm. The system was calibrated using an external standard of hydrolysed and 2AB‐labelled glucose oligomers to create a dextran ladder, as described previously (Royle *et al*., 2006).

### 2.4 Feature analyses

Glycan peaks were pooled based on similar structural or compositional features of the peak glycan members. Features pertaining to a peak were determined based on the major glycan members of that peak based on Saldova *et al*. (2014). (Major structures in each peak and highlighted features are in Table 2.) We performed statistical analysis on glycan peaks (GPs) as well as on pooled features, as described below.

**Table 2:**
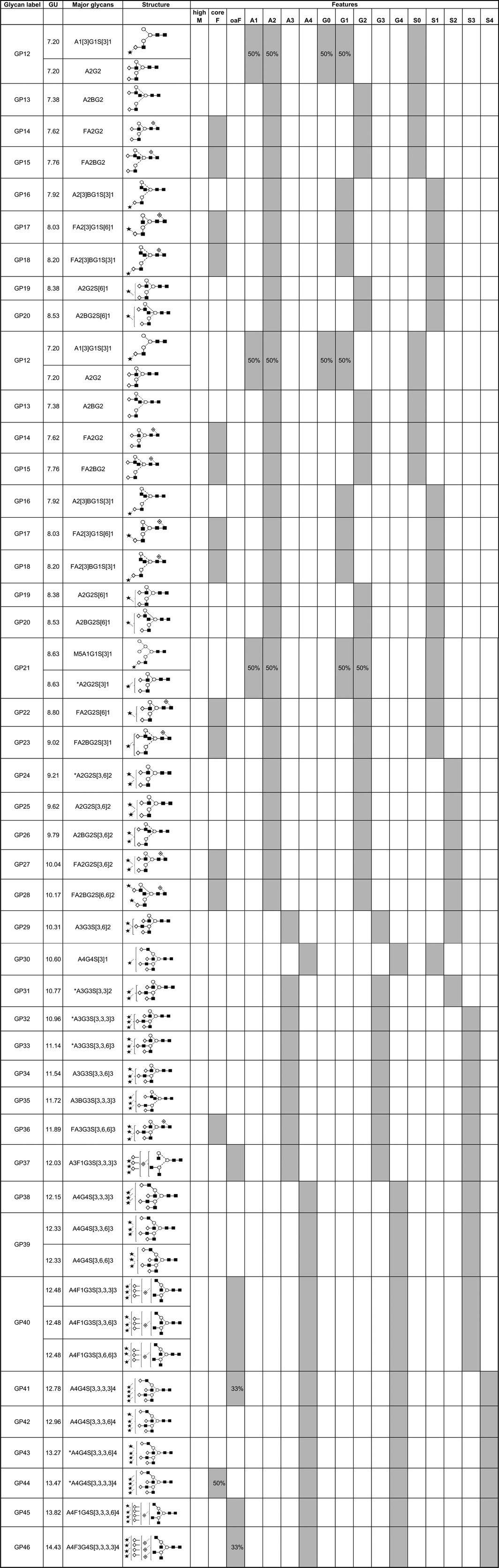
Summary of glycan peaks and their *N*‐glycan composition. Only predominant *N*‐glycans are pictured. Detailed composition of all other *N*‐glycans from human serum is published in Saldova *et al*. (2014). Structure abbreviations: all *N*‐glycans have two core GlcNAcs; F at the start of the abbreviation indicates a core fucose α1,6‐linked to the inner GlcNAc; Mx, number (x) of mannose on core GlcNAcs; Ax, number of antenna (GlcNAc) on trimannosyl core; A2, biantennary with both GlcNAcs as β1,2‐linked; A3, triantennary with a GlcNAc linked β1,2 to both mannose and the third GlcNAc linked β1,4 to the α1,3 linked mannose; A4, GlcNAcs linked as A3 with additional GlcNAc β1,6 linked to α1,6 mannose; B, bisecting GlcNAc linked β1,4 to β1,3 mannose; Gx, number (x) of β1,4 linked galactose on antenna; F(x), number (x) of fucose linked α1,3 to antenna GlcNAc; Sx, number (x) of sialic acids linked to galactose. Dx: isoforms with different mannose‐binding. *Sialic acids isomers (same composition but different sialic acid linkage arrangements resulting in different GUs from the original structures). Peaks calculated into specific features are highlighted in grey (where there is 33 or 50%, it means that the glycans with the given feature are approximately that abundant in the given peak)

### 2.5 Statistical analyses

Glycan UPLC–HILIC data represent the relative percentage areas derived from the HILIC profiles. Therefore, the data are compositional and convey the relative amounts of glycan structures in a sample rather than the absolute quantities with the values sum to a constant value such as 1 or 100%. Serum GP values consisted of 46 peak area values, with additional GP values added based on 17 glycan groups, making 63 GP values in total being analysed. To reduce effects of extreme values and to map the data onto a more Gaussian distribution, GP values were logit‐transformed using log[GP/(100%−GP)] and values for C‐reactive protein (CRP) transformed using log(1 + CRP).

Change in serum glycan values between two time points were analysed using paired *t*‐tests, and false discovery rate (FDR) was computed using the R package *fdrtool*. Relation between GP and CRP was tested using a linear model (R function *aov*) with patient id and time point included as covariates, and Benjamini–Hochberg FDR‐computed as *fdrtool* could not reliably be fitted. Change in CRP over time was analysed using ANOVA (R: *aov*) with patient id and time point as covariates, and Tukey's test for pairwise differences, with pairwise Wilcoxon tests used for control analyses. Throughout, FDR and adjusted p‐values below 5% were considered statistically significant.

## 3 Results

**Figure 2:**
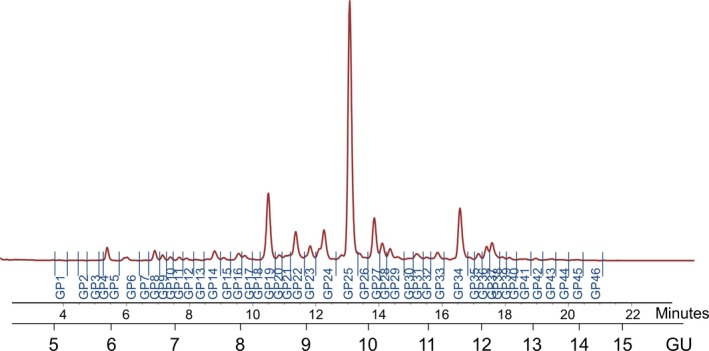
Example of breast cancer sample HILIC–UPLC chromatogram and separation into 46 peaks: GP1–GP46 (listed in Table 1).

The total serum *N*‐glycome was released from all samples and was separated into 46 peaks according to Saldova *et al*. (2014) (Fig. 2). All glycans and derived glycan features were assigned in Saldova *et al*. (2014). The major glycans in each peak, selected in Haakensen *et al*. (2016), are summarized in Table 2.

**Figure 3:**
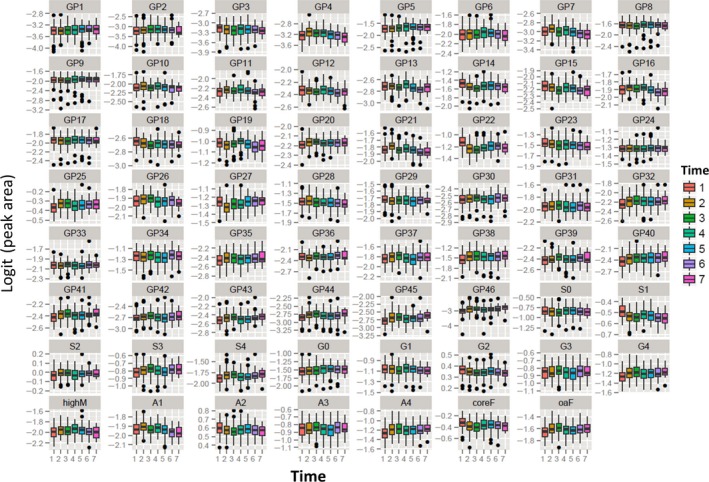
Boxplots of logit‐transformed values of peak percentage areas plotted in each treatment point. Boxes represent the 25th and 75th percentiles with the median indicated by the horizontal line inside the boxes. The whisker bars indicate the 10th and 90th percentiles.

All time points were compared in all patients and in responders and nonresponders separately. Results are summarized in Fig. 3, Table 3, Table S1 and FDR values are in Table S2. Glycan alterations between control versus patients with breast cancer were taken from Saldova *et al*. (2014).

**Table 3:**
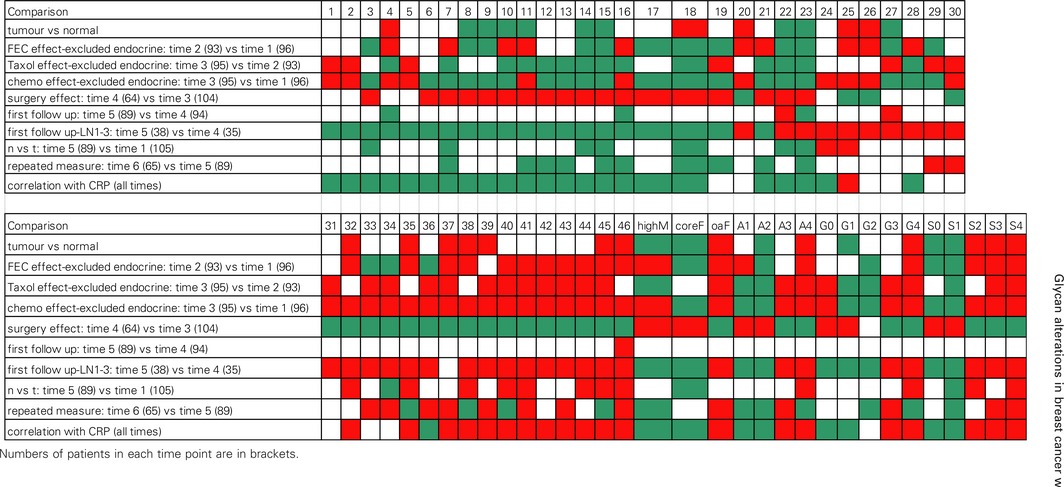
Statistically significant differences between major groups of patients at different treatment times. Predominant glycans in each peak are in Table 2. Comparison of tumour versus normal *N*‐glycome and calculation of the peaks into specific features based on total high mannose glycans, sialylation, galactosylation, branching and fucosylation were taken from Saldova *et al*. (2014). 1‐46 = GP1‐GP46, highM=high mannosylated glycans; coreF=core‐fucosylated glycans; oaF=outer arm fucosylated glycans; A1, A2, A3, A4 =  mono‐, bi‐, tri‐ and tetraantennary glycans; G0, G1, G2, G3, G4 = non‐, mono‐, di‐, tri‐ and tetragalactosylated glycans; S0, S1, S2, S3, S4 = non‐, mono‐, di‐, tri‐ and tetrasialylated glycans. Peaks highlighted in red are found to be increased and in green decreased

### 3.1 Chemotherapy significantly alters *N‐*glycans towards malignant phenotype

For all patients, the serum profile at time 2 was compared to time 1 to elucidate effect of FEC and time 3 to time 2 to elucidate the effect of taxane therapy. Time 3 versus time 1 was compared to look at the effect of total chemotherapy. Most of the GPs were altered after these treatments.

The serum glycan profile after FEC treatment was similar to the profile seen in patients with breast cancer compared with healthy women except for the peaks containing core‐fucosylated bisected biantennary monogalactosylated glycans (peaks 10 and 18) and high mannosylated glycans (peak 11) (Table 3). Additional peaks were found significant with FEC treatment that were not seen in patients with breast cancer, such as increases in peaks containing monoantennary (feature A1), bisected biantennary monogalactosylated (peaks 7 and 16), hybrid, biantennary digalactosylated monosialylated (peak 21), core‐fucosylated bisected biantennary digalactosylated disialylated glycans (peak 28) and tetraantennary, tri‐ and tetragalactosylated, tri‐ and tetrasialylated glycans some with outer arm fucose (peaks 40–44) and decreases in peaks containing monoantennary monogalactosylated (peak 3), core‐fucosylated biantennary monogalactosylated monosialylated (peak 17), biantennary digalactosylated monosialylated (peak 19), triantennary trigalactosylated di‐ and trisialylated some with core fucose (peaks 29, 33, 34 and 36) and total digalactosylated glycans (feature G2) (Table 3).

The effect of FEC treatment differed between patients with complete response and no response despite similar samples sizes (Tables S1 and 4). Nonresponders lacked an increase in certain bisecting glycans as well as highly branched glycans with or without outer arm fucose (Table S1). The number of ER‐negative tumours was too small to stratify the analysis on ER status (Table 4).

**Table 4:** Number of samples in each group

Time point	Response			Lymph node involvement		ER status (responders)		Bevacizumab use		Endocrine		aTotal
	Responders	Only complete	Nonresponders	LN0	LN1–3	ER negative	ER positive	Yes	No	Yes	No	
1	77	19	22	36	39	39	63	50	53	8	96	105
2	75	19	21	35	38	14	61	49	52	9	93	102
3	76	19	22	38	37	13	63	51	52	9	95	104
4	70	18	20	35	35	14	56	47	46	6	88	94
5	55	10	28	35	38	10	45	47	42	8	81	89
6	39	8	21	24	30	8	31	32	33	7	58	65
7	12	1	11	8	16	2	10	15	11	2	24	26

a The total is not exact match as some of the samples had unknown status in one or more features.

Interestingly, the peak containing core‐fucosylated triantennary trigalactosylated trisialylated glycans (peak 36) was found to be increased in patients with no lymph nodes involved, whereas the same peak was found to be decreased in the other patient groups – patients with lymph node‐positive disease, responders and nonresponders and ER‐positive patients (Table S1).

In case of the taxane treatment, again, these alterations in glycan profiles after treatment compared with that before treatment resemble the profiles in women with breast cancer as opposed to healthy women. The exceptions were peaks containing biantennary bisected (peak 4), core‐fucosylated bisected biantennary monogalactosylated monosialylated (peak 18) and core‐fucosylated biantennary digalactosylated disialylated glycans (peak 27) (Table 3). Additional peaks were found significant with taxane treatment, such as an increase in peaks containing simple monoantennary glycans with or without core fucose (peaks 1 and 2), core‐fucosylated biantennary (peak 5), biantennary digalactosylated monosialylated (peak 19), triantennary and trigalactosylated (peaks 29, 31, 33, 34 and 36, and feature A3 and G3), tetraantennary, tri‐ and tetragalactosylated, tri‐ and tetrasialylated glycans some with outer arm fucose (peaks 30, 40–44) and agalactosylated glycans (feature G0) and a decrease in peaks containing monoantennary glycans (feature A1) including monoantennary monogalactosylated glycans some hybrid (peaks 7 and 21), biantennary glycans mostly bisected, mono‐ and digalactosylated, some monosialylated and some core‐fucosylated (peaks 7, 12, 13, 16, 21 and 28) (Table 3).

Nonresponders and patients without lymph node involvement had a *N*‐glycome less affected than responders, complete responders and patients with lymph nodes involved (Table S1).

As for the combined effect of chemotherapy (FEC and taxane therapy), these alterations in glycan profiles demonstrate a trend in the direction of women with breast cancer (as opposed to healthy women) similar to the effect of FEC and taxane therapy separately (Table 3).

Nonresponders and ER‐negative patients had a *N*‐glycome less significantly affected than responders and ER‐positive patients (Table S1). This may be due to the sample cohort numbers as there were less ER‐negative patients and nonresponders in the treatment groups (Table 4). However, the cohort numbers of complete responders were similar to those of nonresponders (Table 4) and complete responders (no tumour left after chemotherapy) showed consistent alterations in the serum *N*‐glycome with the other patient groups (Table S1); therefore, the lower significance in nonresponders is likely due to biology rather than statistics.

Interestingly, the peak containing high mannosylated glycans (peak 11) was found to be decreased in patients with no lymph nodes involved, whereas the same peak was found to be increased in the other patient groups (Table S1).

### 3.2 Surgery significantly alters *N*‐glycans towards control (nonmalignant) phenotype

All samples were compared at time 4 versus time 3 to elucidate the effect of surgery on the serum *N*‐glycome. Most of the GPs after surgery were found to be altered in the direction of healthy women (as opposed to women with breast cancer). The exception was a peak containing core‐fucosylated bisected monogalactosylated monosialylated glycans (peak 18), which was found to be increased in both cases (Table 3). Additional peaks were found to be altered after surgery, such as an increase in peaks containing monoantennary glycans (feature A1) including monoantennary monogalactosylated glycans (peaks 3 and 12), some hybrid (peak 21), high mannosylated (peak 6), biantennary glycans some bisected, mono‐ and digalactosylated, some monosialylated and some core‐fucosylated (peaks 12, 13, 16, 17, 19 and 21) and total agalactosylated glycans (feature G0) and decrease in peaks containing triantennary trigalactosylated di‐ and trisialylated, some core‐fucosylated (peaks 31, 33, 34 and 36), tetraantennary, tri‐ and tetragalactosylated, mono‐, tri‐ and tetrasialylated glycans some with outer arm fucose (peaks 30, 40–44) and total trigalactosylated (feature G3) and triantennary glycans (feature A3) (Table 3).

Interestingly, the alteration of GPs after surgery tended to be opposite to the alteration seen after chemotherapy except for peaks containing high mannosylated (peak 11), agalactosylated (feature G0) and biantennary bisected monogalactosylated monosialylated glycans (peak 16) (increased with both) (Table 3).

Nonresponders, that is patients with a tumour still present at the time of surgery, had no significant alterations in the serum *N*‐glycome after surgery, although the trends were similar to those seen in all responders together (Table S1). Complete responders (no tumour left at the time of surgery) showed alterations in the serum *N*‐glycome similar to those seen in partial and nonresponders despite a limited number of patients in this category (Tables S1 and 4). This indicates that the serum *N*‐glycome reflects the body's response to surgery as well as to the removal of the tumour.

### 3.3 Clinical follow‐up significantly shows *N*‐glycans trend towards malignant phenotype in patients with affected lymph nodes

All samples were compared at time 5 versus time 4 to investigate alterations during the first follow‐up. At time 4, the patients had recovered (6 weeks) from surgery and were about to start radiotherapy. Time 5 was sampled 1 year after surgery. Few glycosylation peaks were found to be significantly changed. There was an increase in a peak containing tetraantennary tetragalactosylated tetrasialylated glycans with three outer arm fucoses (peak 46), which has also been found increased in breast cancer, and there was decrease in peaks containing biantennary bisected monogalactosylated monosialylated glycans (peak 16) and core‐fucosylated biantennary digalactosylated monosialylated glycans (peak 23), the latter was also found to be decreased in cancer (Table 3). One peak containing bisected biantennary glycans (peak 4) was found to be decreased (it increased in cancer) and peaks containing core‐fucosylated biantennary digalactosylated mono‐ and disialylated glycans increased (peaks 22 and 27) (usually decreased in cancer) (Table 3).

There was no significant change in glycosylation in responders, complete responders and patients with no lymph nodes involved (Table S1). There were few changes in nonresponders and a massive alteration of the glycan profile in patients with lymph nodes involved (Tables 3 and S1). The whole glycan profile was altered significantly except for the peak 37 (A3F1G3S[3,3,3]3) (Table 3). The majority of changes were consistent with promalignant phenotype except for peaks containing core‐fucosylated bisected biantennary monogalactosylated monosialylated glycans (peak 18) (increased in cancer, decreased in follow‐up after radiotherapy), and core‐fucosylated biantennary digalactosylated mono‐ and disialylated some bisected glycans (peaks 22, 23 and 27) (decreased in cancer and increased in follow‐up radiotherapy) (Table 3). These significances were not likely to be due to sample numbers in the groups, as the lymph node groups were rather similar in size and numbers of responders are even higher (Table 4). It rather indicates that the follow‐up alters into malignant phenotype only in patients with involved lymph nodes. Also, patients with lymph node‐positive disease may have received a more heavy radiation procedure, with the apical part of the lung and the lymph nodes in the lower part of the neck exposed to such therapy. This is the case for all patients with positive nodes, and all the patients with initial tumour size > 5 cm (locally advanced breast cancer).

Samples at times 5 and 6 (1 and 1.5 years postsurgery and radiotherapy) were compared to time 1 (at the diagnosis).

Comparing the time 5 with time 1, most peaks had a trend in the direction of women with breast cancer (as opposed to healthy women) except for the peak containing core‐fucosylated biantennary bisected monogalactosylated monosialylated glycans (peak 18) (Table 3). Other alterations observed were an increase in peaks containing biantennary digalactosylated disialylated glycans (peak 24) and tetraantennary tri‐ and tetragalactosylated tetrasialylated glycans some with outer arm fucose (peaks 40, 41 and 44) and a decrease in peaks containing monoantennary monogalactosylated (peak 3), biantennary bisected monogalactosylated (peak 7) and triantennary trigalactosylated trisialylated glycans (peak 34) (Table 3).

Comparing time 6 with time 5 reflects changes in the *N*‐glycome over time. We observed a change in several peaks indicating promalignant changes such as increases in peaks containing triantennary trigalactosylated trisialylated outer arm fucosylated (peak 37), tetraantennary tetragalactosylated tri‐ and tetrasialylated, some outer arm fucosylated glycans (peaks 39 and 46) and total trisialylated (feature S3), tetrasialylated (feature S4) and outer arm fucosylated glycans (feature oaF) and decrease in peaks containing high mannosylated (peak 11, feature highM), core‐fucosylated bisected biantennary digalactosylated, some monosialylated glycans (peaks 15 and 23) and total monosialylated (feature S1) and biantennary glycans (feature A2) (Table 3). Additional peaks were altered including an increase in peaks containing total triantennary trigalactosylated glycans (feature G3 and A3) including di‐ and trisialylated and some core‐fucosylated glycans (peaks 29, 33, 34, 36) and tetraantennary tetragalactosylated mono‐ and tetrasialylated glycans (peaks 30, 41 and 43) and decreases in peaks containing total monoantennary glycans (feature A1) including monogalactosylated monosialylated and hybrid glycans (peaks 12, 16 and 21), biantennary mono‐ and digalactosylated, some monosialylated, some bisected (peaks 7, 12, 13, 16, 19 and 21), tetraantennary trigalactosylated trisialylated outer arm fucosylated (peak 40) and total digalactosylated glycans (feature G2) (Table 2). Opposite changes were also found such as decreases in peaks including core‐fucosylated bisected biantennary monogalactosylated monosialylated (peak 18), triantennary bisected trigalactosylated trisialylated (peak 35) and total tetragalactosylated (feature G4) and tetraantennary glycans (feature A4) including tri‐ and tetrasialylated and some outer arm fucosylated glycans (peaks 38 and 45) (increased in cancer) (Table 3).

### 3.4 CRP correlates with malignant phenotype

**Figure 4:**
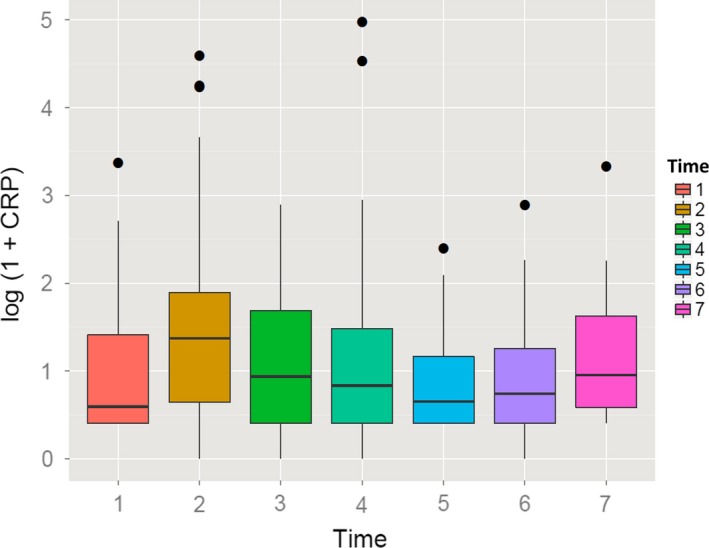
Boxplots of log CRP plotted in each treatment point. Boxes represent the 25th and 75th percentiles with the median indicated by the horizontal line inside the boxes. The whisker bars indicate the 10th and 90th percentiles.

CRP was positively correlated with those GPs that have been found associated with malignant phenotype (Table 3). CRP significantly increases with FEC treatment, then decreases and reaches lowest at 1 year after surgery and then starts to increase one and two years after surgery (Fig. 4).

## 4 Discussion

### 4.1 Glycosylation patterns significantly distinguish the effects of chemotherapy, surgery and radiation

Previously, increases in sialylation, branching, outer arm fucose (which is part of sLex) and decrease in high mannosylated and biantennary core‐fucosylated glycans were found in patients with breast cancer compared with healthy controls (Marino *et al*., 2012; Saldova *et al*., 2014).

We found sialylation to be increased (S2–S4 versus S0–S1), after chemotherapy, in follow‐up after radiotherapy in lymph node‐positive patients and decreased after surgery (Table 3, Fig. 3). Galactosylation also increased (G3–G4 versus G0–G2) after chemotherapy, in follow‐up after radiotherapy in lymph node‐positive patients and decreased after surgery (Table 3, Fig. 3). Branching (A3–A4 versus A1–A2) had the same trend as sialylation and galactosylation, being increased after chemotherapy, in follow‐up after radiotherapy in lymph node‐positive patients and decreased after surgery (Table 3, Fig. 3).

Increase in sialylation, branching and outer arm fucose (sLex) was found in inflammatory conditions (Marino *et al*., 2012) and correlates with an increase in inflammation, metastasis, drug resistance and tumour volume and progression (Marino *et al*., 2012; Saldova *et al*., 2013).

We found core fucose to be decreased in cancer, after chemotherapy and in follow‐up after radiotherapy in lymph node‐positive patients and increased after surgery. Outer arm fucose was found to be increased in cancer, after chemotherapy and in follow‐up after radiotherapy in lymph node‐positive patients and decreased after surgery (Table 3, Fig. 3). Fucose attached to the core and to the outer arm tends to occur in opposite situations. Indeed, Lauc *et al*. (2010) showed that hepatocyte nuclear factor 1a (HNFα) regulates fucose synthesis pathways leading to core‐fucosylated versus outer arm fucosylated glycans. The role of HNF1α in the regulation of core versus outer arm fucosylation may be the molecular mechanism behind the reported association between common variants of HNF1α and inflammatory markers such as CRP as well as several diseases where inflammation plays a key pathogenic role such as cancer (Lauc *et al*., 2010). Core fucose regulates antibody‐dependent cellular cytotoxicity activity, growth factor receptor signalling and function of adhesion molecules (Miyoshi *et al*., 2008).

Total high mannose glycans/M6 (major component of peak 11, obvious smaller part of peak 6) decreased in cancer, increased after chemotherapy and surgery and decreased again at 2 years postsurgery (Table 3, Fig. 3). Mannosylation was found to be decreased in cancer and with progression of inflammation (Gornik *et al*., 2007; Saldova *et al*., 2014).

Particular glycans that show interesting trends with treatment and may be connected to cancer biology are hexamannosylated glycans (M6, peak 11) and core‐fucosylated triantennary trigalactosylated trisialylated glycan (FA3G3S[3,6,6]3, peak 36).

Hexamannosylated glycan (M6 D3, peak 11) decreased in breast cancer. This glycan increased after FEC, but decreased after taxane therapy (Table 3, Fig. 3). When looking at total effect of chemotherapy, it was found to be increased except for patients with no lymph node involvement, where it was found to be decreased. It was found to be increased after surgery and decreased in follow‐up after radiotherapy in lymph node‐positive patients and in further follow‐ups. Haakensen *et al*. (2016) observed that this glycan is correlated with decreased mitochondrial fatty acid beta oxidation. Decrease in this glycan in cancer indicates an increase in cell energy production.

Core‐fucosylated triantennary trigalactosylated trisialylated glycans (FA3G3S[3,6,6]3, peak 36) are not significantly affected in cancer, but the peak decreases after FEC treatment in lymph node‐positive patients, increases after taxane and total chemotherapy, decreases after surgery, increases in follow‐up, most prominently in lymph node‐positive patients (Table 3, Fig. 3, Table S1). In previous studies, we have found that it strongly correlates with increased BMI and is associated with decreased integrin‐mediated cell adhesion and decreased adipocyte activity (Haakensen *et al*., 2016). An increase in BMI reduces adipocyte activity and increases inflammation and the risk of cancer (van Kruijsdijk *et al*., 2009). Decrease in cell adhesion could indicate increased mobility of tumour cells (Haakensen *et al*., 2016), which may explain why this glycan behaves differently in lymph node‐positive and lymph node‐negative patients after FEC.

We found strong correlation of glycosylation and the inflammatory marker CRP (Table 3). CRP increased in cancer including breast cancer, it is a prognostic indicator, and its increased levels indicate more aggressive disease, higher risk of recurrence and reduced survival (Asegaonkar *et al*., 2015; Ham and Moon, 2013). Chronic inflammation is a well‐known feature of cancer microenvironment, and it contributes to the development and progression of cancer (Asegaonkar *et al*., 2015; Ham and Moon, 2013). Mills *et al*. (2008) found that chemotherapy increases certain markers of inflammation, particularly markers of endothelial and platelet activation, but CRP was not significantly changed. CRP significantly increased after FEC treatment and started to decrease after taxane therapy and reaching a minimum 1 year postsurgery, then increasing again (Fig. 4). Glycosylation took a pro‐inflammatory direction after chemotherapy, then anti‐inflammatory after surgery and then back pro‐inflammatory after radiation and follow‐up. We observed a significant difference between CRP and glycosylation after taxane therapy, where the glycosylation pattern was still significantly pro‐inflammatory, but the CRP starts to have a decreasing trend (Fig. 4). Perhaps, there is a change in inflammatory response, switch from acute to chronic inflammation, where acute phase response to chemotherapy measured by CRP starts to decrease, whereas chronic inflammatory response reflected by glycosylation profile still persists. Surgery had a significant anti‐inflammatory effect on glycosylation, but CRP was not significantly altered. The lowest level of CRP was 1 year postsurgery, whereas the most anti‐inflammatory *N*‐glycome was after surgery (Fig. 4). It seems like that the acute inflammation continues to a decrease in the first follow‐up before starting to increase again 2 years after surgery, whereas chronic inflammation starts to increase in the first follow‐up and keeps increasing in other follow‐ups. Piperis *et al*. (2012) have shown that radiotherapy did not significantly change CRP levels; however, when two treatment groups were compared both treated with chemotherapy but only one was treated radiotherapy, the radiotherapy‐treated group showed higher levels of CRP compared to untreated group (Kilicaslan *et al*., 2014). We see increases in pro‐inflammatory *N*‐glycome in follow‐up after radiotherapy, but only in lymph node‐positive patients and CRP levels decreased significantly when compared to the levels after taxane therapy, but not significantly comparing to the previous time point before radiotherapy.

### 4.2 Glycan alterations after chemotherapy and in follow‐up are due to its effect on host immune system and feedback to the tumour microenvironment

Chemotherapy and radiotherapy have profound effects on the immune system. While the numbers of white blood cells and lymphocytes decreased in patients with breast cancer, NK cytotoxicity and intracellular T‐cell signalling cytokines (IFN‐γ, IL‐4 and IL‐2) were higher at baseline comparing to healthy controls, and increased during and after 5‐fluorouracil, epirubicin and cyclophosphamide (FEC) treatment (Mozaffari *et al*., 2009). The NK cytotoxicity and cytokines were higher in patients who received both chemo‐ and radiotherapy than in those who received only radiotherapy (Mozaffari *et al*., 2009).

A tumour‐associated inflammation can be initiated by cancer therapy, surgery results in activation of infection‐ or stress‐sensing pathways, and radiation and chemotherapy cause massive necrotic death of cancer cells and surrounding tissues, which in turn trigger an inflammatory reaction which can enhance the cross‐presentation of tumour antigens and subsequent induction of an antitumour immune response (Grivennikov *et al*., 2010). This beneficial host inflammatory response is triggered by necrosis through the activation of TLR4 and production of pro‐inflammatory cytokine IL‐1β (Ghiringhelli *et al*., 2009). On the other hand, chemotherapeutic drugs such as paclitaxel, 5‐fluorouracil and doxorubicin induced inflammation. Introduction of pro‐inflammatory cytokines may lead to treatment failure through an increase in angiogenesis, proliferation and metastasis (Vyas *et al*., 2014). Anti‐inflammatory drugs may be used in combination to kill the cancer cells, but are not cytotoxic for normal cells and have been found to decrease cancer incidence if used as prophylactics (Grivennikov *et al*., 2010).

Cancer and chronic inflammation are associated with similar changes in the serum *N*‐glycome especially increased sialylation, branching and outer arm fucosylation. The serum *N*‐glycome is probably affected by certain immunological triggers secreted by the tumour microenvironment, where liver and plasma cells are important targets of these factors (Hamfjord *et al*., 2015). The glycoproteins secreted by the liver and plasma cells contribute to the majority of glycoforms present in serum (Hamfjord *et al*., 2015), so what we see is a reflection of the host response rather than tumour glycosylation.

The liver produces acute phase glycoproteins, including CRP, that increase in concentration during inflammation as a result of liver hepatocytes being stimulated by pro‐inflammatory cytokines (Arnold *et al*., 2008). Highly sialylated and branched glycans and glycans with sLex produced on these acute phase glycoproteins stay longer in circulation and have anti‐inflammatory properties, and they may therefore affect cancer spread and metastasis (Saldova *et al*., 2008). Agalactosylated glycoforms of IgG (lower galactosylation and sialylation) have increased complement‐dependent cytotoxicity, alter the immune response in patients with cancer and increase with cancer progression (Arnold *et al*., 2008; Hamfjord *et al*., 2015). After chemotherapy and in follow‐up in lymph node‐positive patients, we observed increased galactosylation and sialylation, indicating an increase in inflammation.

The inflammatory cytokines also stimulate the expression of the glycosyltransferases in tumour cells (Arnold *et al*., 2008), which also add to the alterations in glycosylation. Indeed, certain glycoforms present in serum are derived from tumour cells, for example RNase 1 for pancreatic and PSA for prostate cancer, but in far lower amounts (Hamfjord *et al*., 2015).

### 4.3 Glycan alterations after surgery reflect removal of tumour and possible effect of the surgery

Glycosylation changes after surgery are rather anti‐inflammatory, which is in agreement with removal of the tumour or possible tumour‐dormant cells in the microenvironment and therefore removal of the inflammatory trigger as well as a possible effect of the surgery.

### 4.4 Follow‐up glycosylation changes show promalignant phenotype especially in lymph node‐positive patients

Most patients underwent radiotherapy after chemotherapy and surgery. Patients with lymph node‐positive disease have a *N*‐glycome more affected after taxane therapy than patients with no lymph nodes affected. This may indicate that taxane therapy is more effective in patients with lymph node‐positive disease. Indeed, response to taxane therapy was similar for node‐negative and node‐positive patients, although somewhat higher percentage of node‐positive patients responded (Green *et al*., 2005).

Glycosylation profiles during clinical follow‐up were rather in line with promalignant changes although less pronounced, changes were especially less noticeable as the time progressed. In the first follow‐up, approximately 10 months after radiotherapy, there were a few changes in nonresponders, but no significant changes in responders. In case of lymph node‐positive patients, there was a massive change in glycosylation but not in patients without lymph node involvement. These changes probably reflect the above‐described mechanism linked to immune response.

### 4.5 Responders show bigger glycosylation changes with chemotherapy and surgery than nonresponders

Nonresponders have less affected *N*‐glycome than responders with chemotherapy and surgery. Serum *N*‐glycome tends to be more affected by the chemotherapy treatment in women with lymph node‐positive disease and in responders (Table S1), which could indicate that the treatment is working resulting in reduced tumour burden and such reduced immune response to it in responders and affecting the system biology of the patients especially these where the tumour is disseminated (lymph node‐positive disease). It could also partially reflect the host reaction to treatment. Complete responders (no tumour left at the time of surgery) show alterations in the serum *N*‐glycome after surgery similar to those seen in partial and nonresponders despite a limited number of patients in this category (TablesS1 and 4). This indicates that the serum *N*‐glycome reflects the body's response to surgery as well as to the removal of the tumour. Glycosylation changes in serum *N*‐glycome could therefore serve as biomarker for response to treatment, particularly neoadjuvant chemotherapy and surgery (Casey *et al*., 2016).

## 5 Conclusions

We here present the first profiling of serum *N*‐glycans in patients with breast cancer, following each patient from time of diagnosis, throughout neoadjuvant treatment, surgery, radiotherapy and clinical follow‐up. The glycan alterations identified reflect the host response to treatment as well as the tumour and suggest a shift from acute to chronic inflammation during cancer treatment. This preliminary study should be repeated in future in independent cohorts and different populations. Further study could also include identifying tumour‐specific glycosylation changes with treatment from the tissues at different time points.

## Author contributions

ALBD and PMR conceived and initiated this study. RS, VDH, ALBD and PMR designed the experiments. VDH and OE collected the clinical samples and provided clinical information. RS and HS carried out the experiments and generated the results. RS, VDH, ER and IW conducted the statistical analysis. RS and VDH interpreted the results and drew the conclusions. RS with significant input from VDH wrote the initial draft of the manuscript which was reviewed by all coauthors.

## Supporting information


**Table S1.** Statistically significant differences among all groups of patients at different treatment times.

**Table S2.** Statistically significant differences between major groups of patients at different treatment times including FDR values.
