# Supplementary material for: Serum N‐glycome alterations in breast cancer during multimodal treatment and follow‐up
Source: Mol Oncol. 2017 Jul 24;11(10):1361–79. doi: 10.1002/1878-0261.12105 (PMC5623820; doi:10.1002/1878-0261.12105)
Supplement: Supplementary file 3 [file MOL2-11-1361-s003.docx]

**Supporting Information**

**Supplementary Table S1. Statistically significant differences among all groups of patients at different treatment times**

Peaks highlighted in red are increased and in green are decreased.

**Supplementary Table S2. Statistically significant differences between major groups of patients at different treatment times including FDR values**

Peaks highlighted in red are increased and in green are decreased.
